# Solid variant of aneurysmal bone cyst in the tibia treated with simple curettage without bone graft: a case report

**DOI:** 10.1186/1477-7819-10-45

**Published:** 2012-02-20

**Authors:** Rumi Takechi, Takashi Yanagawa, Tetsuya Shinozaki, Toshio Fukuda, Kenji Takagishi

**Affiliations:** 1 Department of Orthopaedic Surgery, Gunma University Graduate School of Medicine. 3-39-22, Showa, Maebashi, Gunma, 371-8511, Japan; 2 Department of Laboratory Sciences & Pathology, Gunma University School of Health Sciences, 3-39-22, Showa, Maebashi, Gunma, 371-8511, Japan

**Keywords:** aneurismal bone cysts, curettage, cyclooxygenase 2

## Abstract

The solid variant of aneurysmal bone cyst (solid ABC) is rarely encountered in long bones and appropriate treatment for this disease remains unclear. We experienced a 13-year-old boy suffering from pain in his left knee caused by solid ABC. Simple curettage of the bone lesion without any adjuvant therapy and a bone graft gave immediate pain relief. Histological examination of the surgical specimen showed typical features of solid ABC, and cycloxygenase-2 (COX-2) expression was confirmed in giant cells with a background of spindle cells by immunohistochemistry. Magnetic resonance imaging showed that soft tissue edema surrounding the lesion was improved two months after surgery and there was no indication of recurrence two years after surgery.

If COX-2 secreted from the tumor induces soft tissue edema, simple curettage of the bone lesion seems to be a reasonable treatment for solid ABC and is able to minimize invasive treatment of the patients.

## Background

The term 'solid variant of aneurysmal bone cyst (solid ABC)' was first described by Sanerkin et al. as a non-cystic bone lesion with histological features found in the solid parts of a conventional aneurysmal bone cyst [1]. Meanwhile, Jaffe et al. first reported giant cell reparative granuloma (GCRG) as a reactive lesion to an intraosseous hemorrhage in the jaw [2], and Lorenzo et al. described GCRG in the short tubular bones of the hands and feet [3]. The histological features of solid ABC resembled those of GCRG, which indicated a close relationship between the two conditions [1].

Solid ABC commonly affects the axial skeleton and short tubular bones of the hands and feet, and is rarely encountered in the long bones. Ilaslan et al. reported 30 cases of solid ABC in the long bones, eight of which underwent magnetic resonance (MR) imaging. MR image findings showed solid bone lesions in all eight cases and surrounding edema in 50% [4]. With regard to the treatment, solid ABCs of short tubular bones have been treated with resection, amputation, and curettage supplemented with adjuvant therapies [5-7]; however, it remains unknown which treatment is most appropriate for tumors in long bones. In addition, it is obscure why solid ABC accompanies edematous lesions and how to treat the edema.

Here, we report a case of solid ABC in the left tibia treated by only curettage with radiological evaluation, and examined the cause of edema in solid ABC.

## Case Presentation

**Figure 1:**
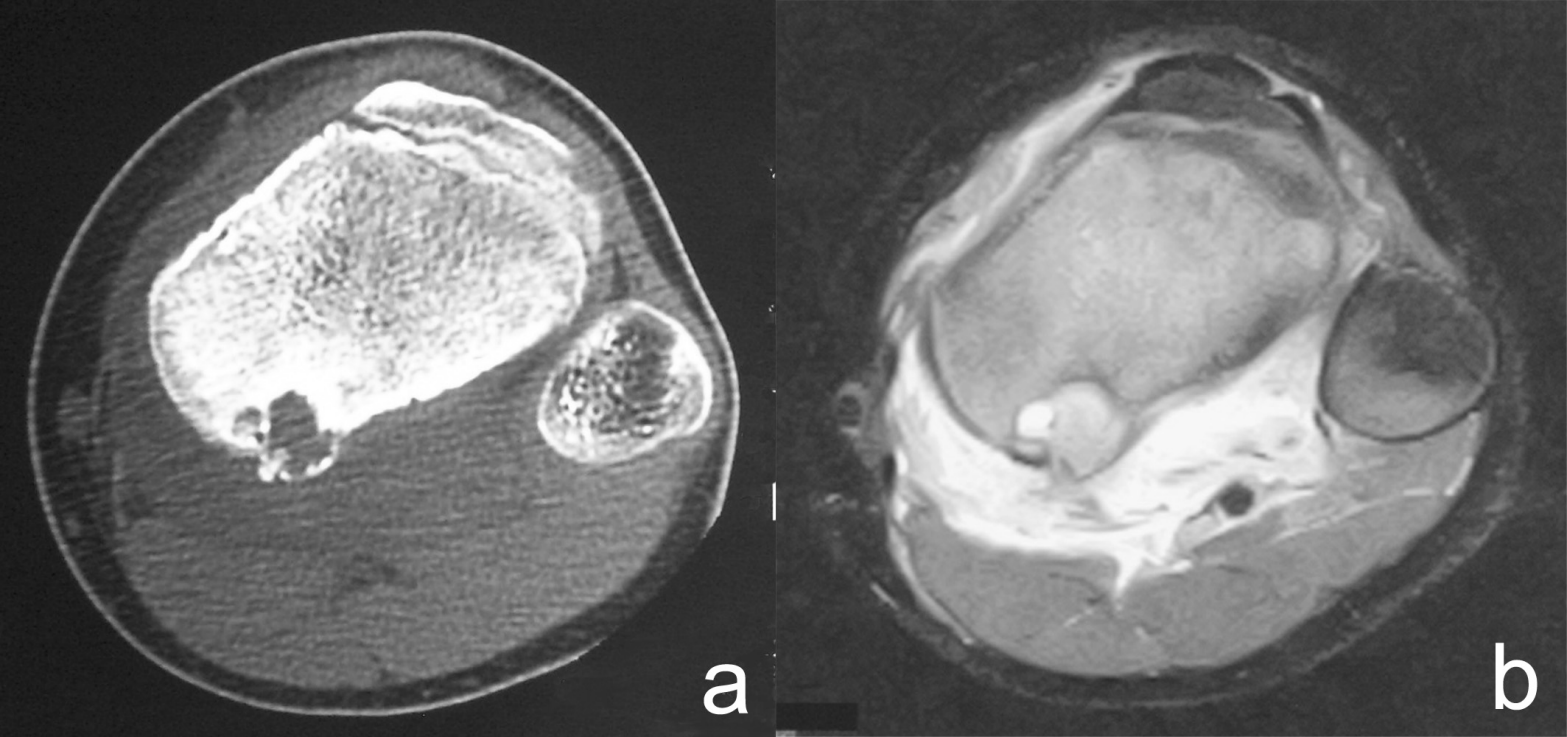
**A 13-year-old boy with a solid ABC in the left tibia**. a. CT showed an osteolytic expansive lesion with cortical thinning. b. The bony lesion showed a mixture of low and high signal intensity on T2-weighted MR images with an edematous lesion adjacent to the bony lesion.

**Figure 2:**
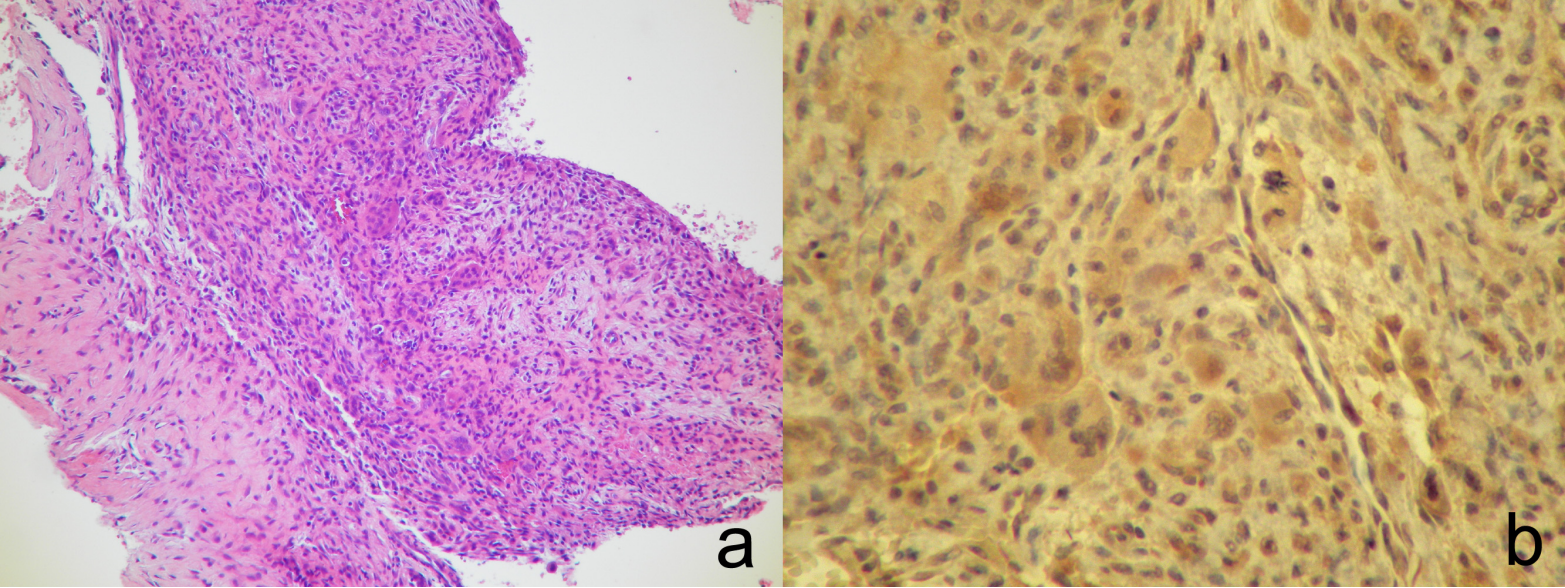
**Photomicrographs showing a) belt-shaped giant cells against a background of spindle cells and scattered osteoclasts**. These features are compatible with solid ABC (hematoxylin-eosin x400). b) Immunohistochemical staining revealed COX-2 expressed in giant cells and spindle cells (x400).

**Figure 3:**
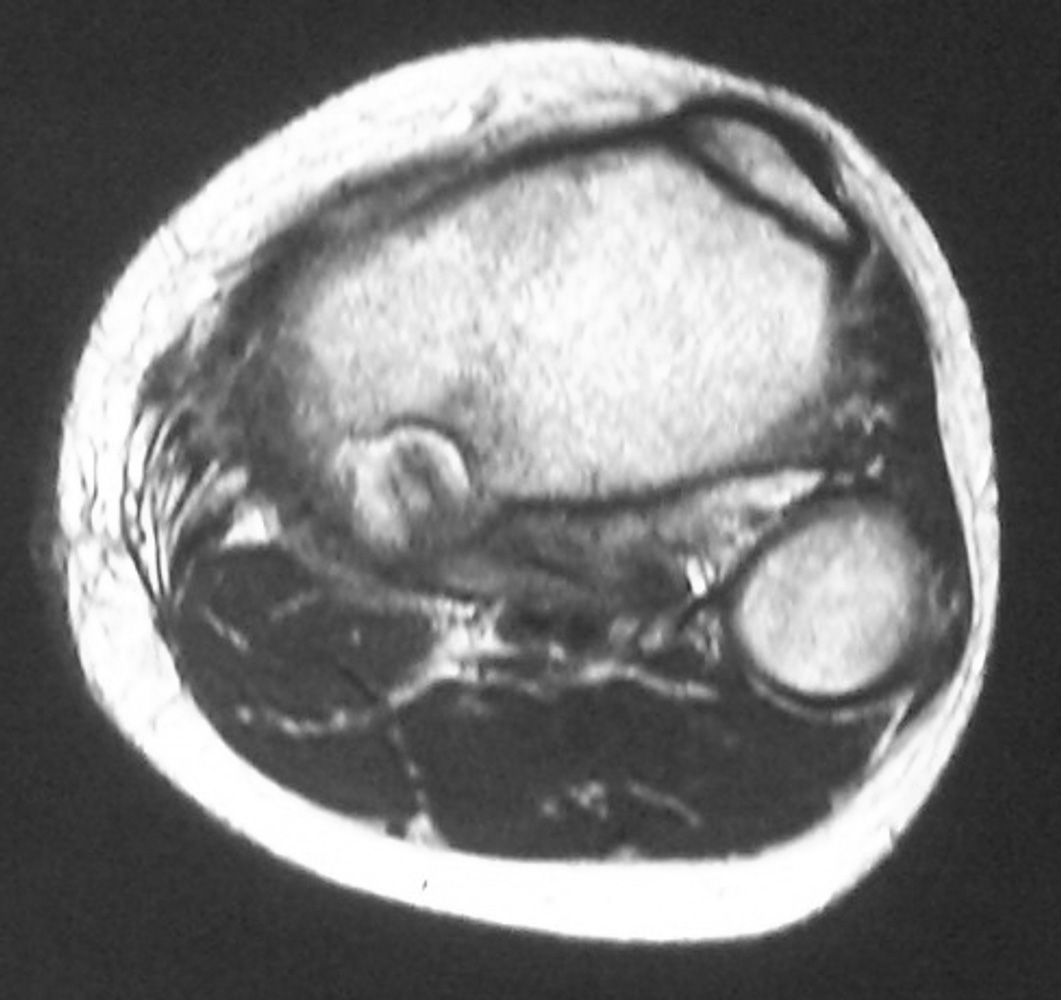
**MR imaging 2 months after surgery**. Soft tissue edema reduced markedly.

**Figure 4:**
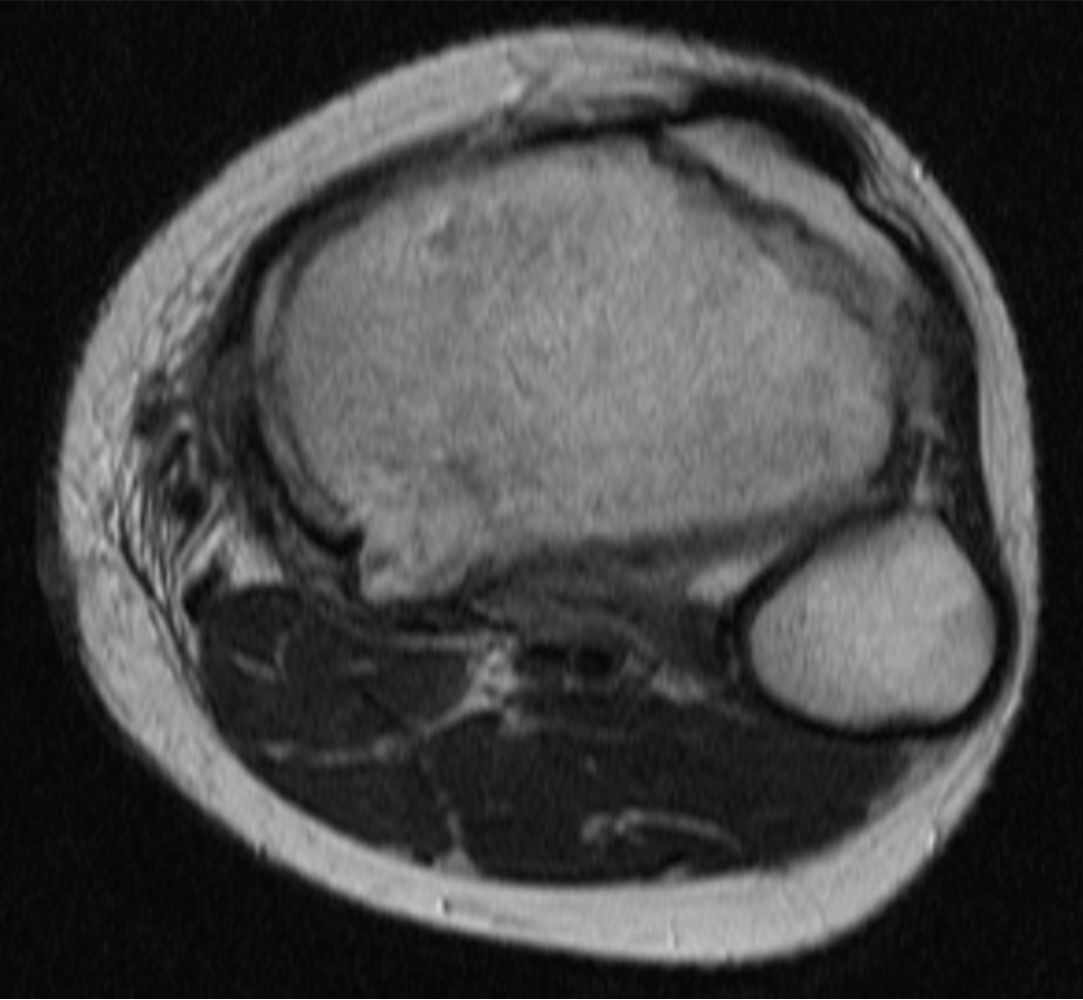
**MR imaging at most recent visit**. There was no tumor recurrence.

**Figure 5:**
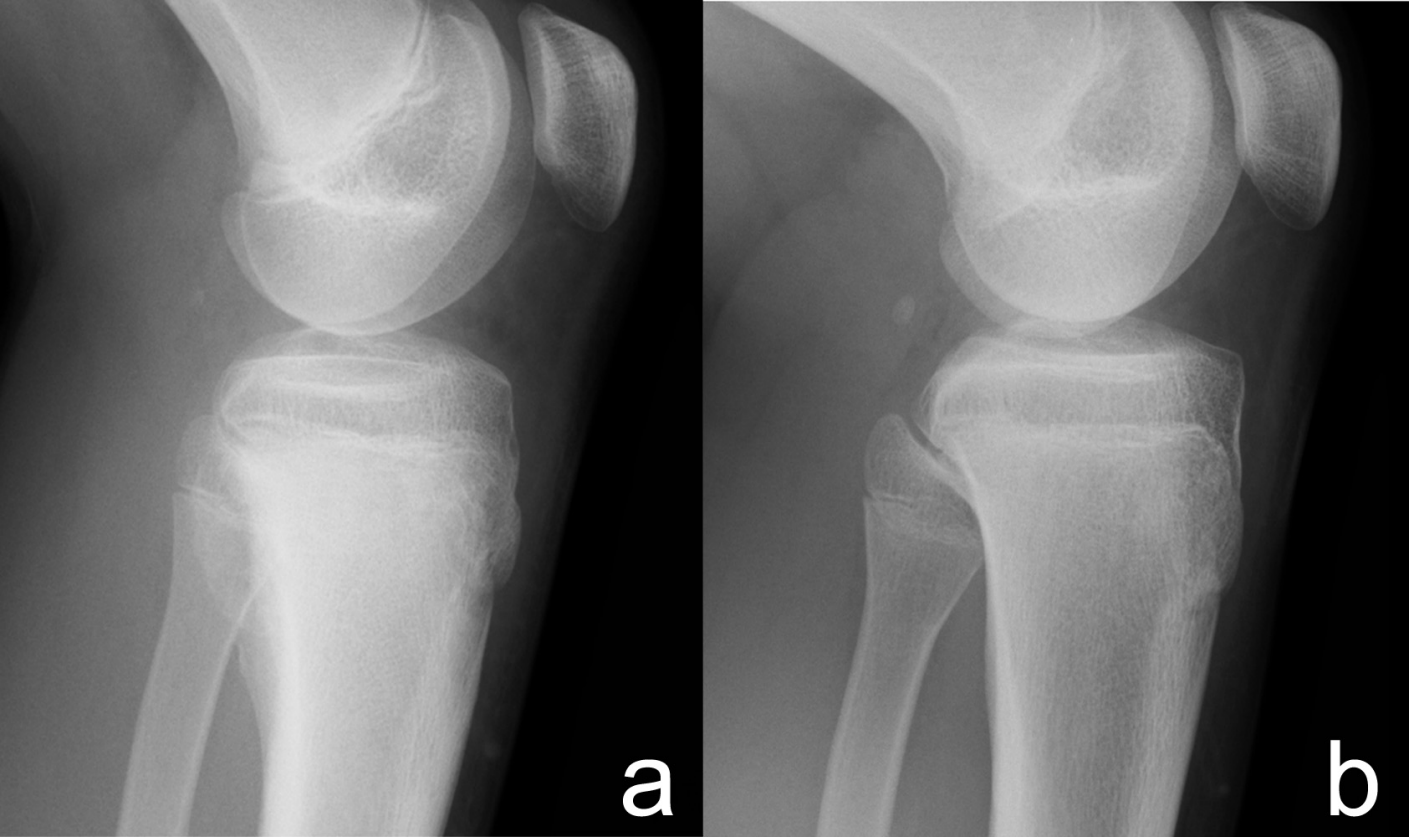
**X-ray findings of the lesion**. Shell-like lesion on the posterior surface of the tibia before operation (a) completely disappeared and the host bone had consolidated well 2 years after operation (b).

A 13-year-old boy was referred to our hospital on July 2007 complaining of slight pain and swelling in his left popliteus. He initially noticed knee pain during knee motion without any major trauma in February 2007 and swelling of his left popliteal fossa two months later. On physical examination, there was swelling without tenderness and local heat of his left posterior knee. Laboratory data were uniformly unremarkable, except for a high level of alkaline phosphatase. Plain radiographs showed an expansive and well-defined osteolytic lesion surrounded by a shell in the cortex of the metaphysis of the left proximal tibia. Computed tomography (CT) of the tibia showed an osteolytic expansive lesion with cortical thinning (Figure 1a). On MR imaging, the bony lesion was iso-intense on T1 with a mixture of low and high signal intensity on T2-weighted images with an edema-like soft tissue lesion adjacent to the bony lesion (Figure 1b). First, needle biopsy of the soft tissue mass was performed because the lesion was suspected to be malignant. Histology revealed that the lesion was fibromuscular with lymphoid tissue without malignancy, and surgical treatment was performed. Intraoperative biopsy of the white soft tissue infiltrating the soleus muscle revealed that the soft tissue lesion was similar to the needle biopsy specimens pre-operation. After the soft tissue was confirmed to be reactive to inflammation and without malignancy, the bone lesion, which was occupied with solid white tissue, was curetted without adjuvant therapy, such as phenol treatment or bone grafting. Histological examination of the surgical specimens showed a solid variant of aneurysmal bone cyst with belt-shaped giant cells against a background of spindle cells and scattered osteoclasts (Figure 2a). Immunohistochemistry revealed cyclooxygenase-2 (COX-2) expressed in not only giant cells but also spindle cells (Figure 2b), which was confirmed by anti-human COX-2 antibody (IBL, Japan). The posterior knee pain immediately disappeared after surgery. On MR imaging at follow-up 2 months after surgery (Figure 3), the soft tissue edema had markedly improved. There was no recurrence of the lesion two years after the operation (Figure 4) and the operated bone exhibited good remodeling (Figure 5a, b)

## Discussion

Solid ABC commonly originates from short bones and rarely affects long bones. The recurrence rate of lesions treated with simple curettage is relatively high, and therefore, amputation or curettage with adjuvant therapy, such as phenol and ethanol, has been applied to solid ABC in the short bones by some authors [5-7]. Oda et al. reported, however, that no patients had local recurrence after curettage or intralesional resection [8].

In the current case, simple curettage of the bony lesion without adjuvant therapy and a bone graft could cure solid ABC in a long bone. After the operation, the surrounding soft tissue edema was markedly relieved and the bony lesion completely remodeled, which was confirmed with post-operative CT and MR images. The cause of soft tissues edema surrounding the bone lesion of solid ABC remains unclear, although edema is one of the features of solid ABC on MR imaging. Some benign tumors with inflammation, such as osteoid osteoma and chondroblastoma, are known to produce prostaglandin, COX-2 and other cytokines related to inflammation [9-11]. In our solid ABC case, COX-2 was expressed in giant cells and spindle cells. If cytokines from the bone lesion of solid ABC induce soft tissue edema, simple curettage of the bony lesion seems to be a better surgery than wide resection, including edematous soft tissues. There is a possibility that the edema was provoked not only by COX-2 from the tumor but also by a small fracture of the shell-like cortex; however, if the cause of his pain and edema was a fracture at the lesion, the symptoms would have been relieved within 2 months. Therefore, we diagnosed that his pain and edema were mainly induced by the tumor.

Recently, we and Hirn et al. reported that simple curettage without bone grafts for benign bone tumors, such as giant cell tumor, chondroblastoma, enchondroma, simple bone cyst, and so on, in long bones gives sufficient bone strength to host bones [12,13]. Our previous study showed that no patients needed orthosis 3 months after curettage for lesions of wide-ranging size (2-196 cm^3^). This indicates that larger solid ABCs than in the current report are expected to be cured with simple curettage. Some authors reported that extensive curettage of the tumor may be more important than the use of adjuvant therapies to prevent local recurrence, which enabled us to use no adjuvant treatment after curettage [14,15].

The typical MR imaging appearance of solid ABC is as follows: a bony lesion is heterogeneous with a predominantly high signal on T2-weighted images and edema adjacent to the lesion was seen in 50% of cases [4]. These findings may lead physicians to misdiagnose this disease as malignant. Radiologists and orthopedic surgeons should keep in mind solid ABC with lytic bone lesions that have soft tissue edema to avoid unnecessary overtreatment.

## Conclusion

We experienced a solid ABC with surrounding soft tissue edema which was presumably induced by COX-2 from the tumor. Simple curettage of a bone lesion secreting COX-2 achieved an excellent result with immediate reduction of the edema and good remodeling of the curetted lesion. To minimize surgical invasion, diagnosis of a lytic bone lesion with a sclerotic rim and surrounding soft tissue edema is important.

## Consent

Written informed consent was obtained from the patient's parents for publication of this Case report and any accompanying images. A copy of the written consent is available for review by the Editor-in-Chief of this journal.

## List of abbreviations

ABC: aneurysmal bone cyst; COX-2: cycloxygenase-2; GCRG: giant cell reparative granuloma; MR: magnetic resonance; CT: computed tomography.

## Competing interests

The authors declare that they have no competing interests.

## Authors' contributions

RT collected data, carried out the immunohistochemistry and drafted manuscript. TY conceived of the design and helped to draft the manuscript. TF contributed to histological diagnosis. TS and KT revised manuscript critically for important intellectual content. All authors read and approved the final manuscript.
